# Supplementary material for: HIV-1 release requires Nef-induced caspase activation
Source: PLoS One. 2023 Feb 13;18(2):e0281087. doi: 10.1371/journal.pone.0281087 (PMC9925082; doi:10.1371/journal.pone.0281087)
Supplement: S1 Table — Note: Samples labeled HIV, QVD, and UI are from experiments with HIV-1BAL whereas samples labeled Nef+ and Nef- represent experiments with HIV-1NL4-3 with or without the gene sequence encoding functional accessory protein, Nef. Samples labeled 1, 2, or 3 correspond to donor. The number of total sequences represent all sequences with reads >0, and they include coding sequences as well as pseudogenes, processed transcripts, Long intergenic non-coding RNAs (LincRNA) and anti-sense sequences. (PPTX) [file pone.0281087.s001.pptx]

## Slide 1
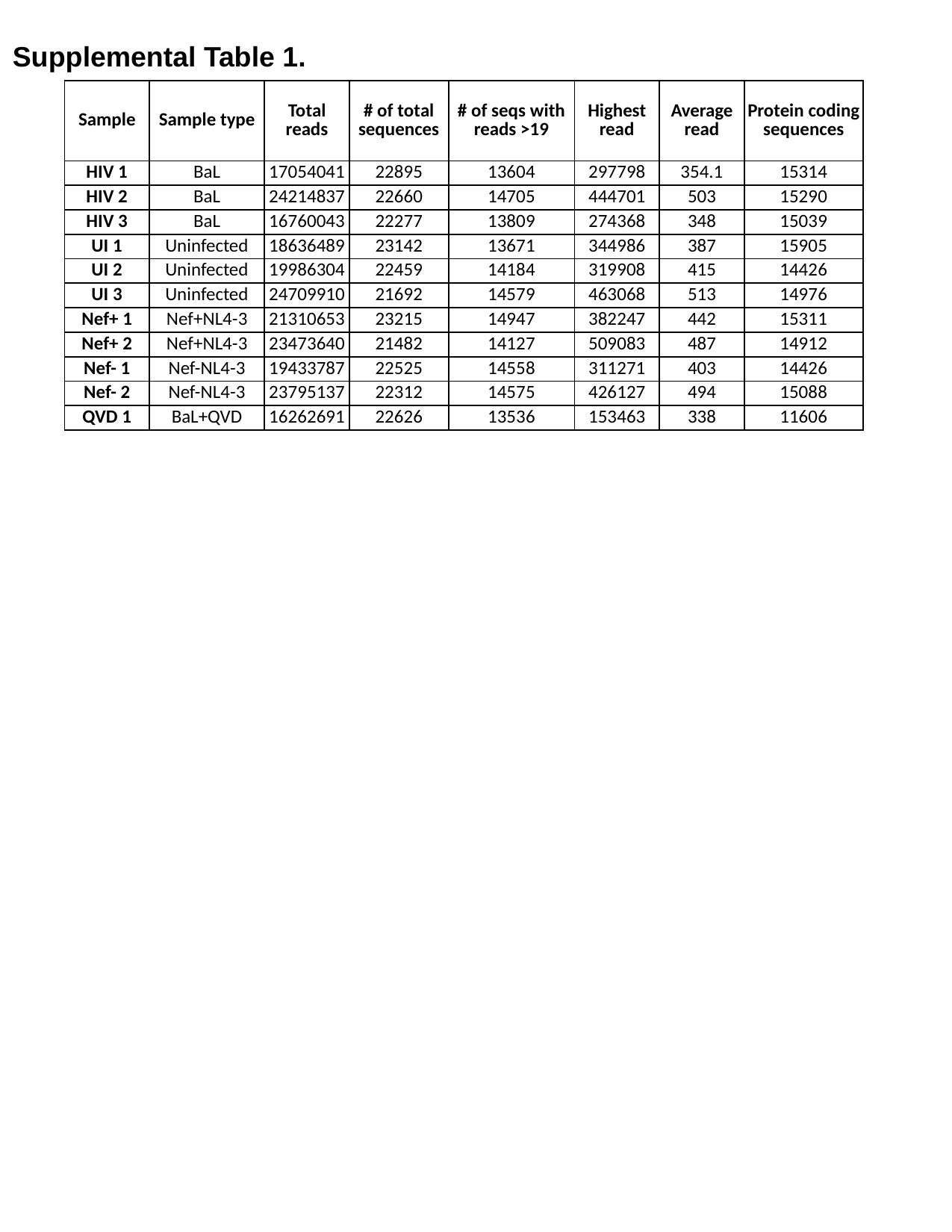

Supplemental Table 1.
| Sample | Sample type | Total reads | # of total sequences | # of seqs with reads >19 | Highest read | Average read | Protein coding sequences |
| --- | --- | --- | --- | --- | --- | --- | --- |
| HIV 1 | BaL | 17054041 | 22895 | 13604 | 297798 | 354.1 | 15314 |
| HIV 2 | BaL | 24214837 | 22660 | 14705 | 444701 | 503 | 15290 |
| HIV 3 | BaL | 16760043 | 22277 | 13809 | 274368 | 348 | 15039 |
| UI 1 | Uninfected | 18636489 | 23142 | 13671 | 344986 | 387 | 15905 |
| UI 2 | Uninfected | 19986304 | 22459 | 14184 | 319908 | 415 | 14426 |
| UI 3 | Uninfected | 24709910 | 21692 | 14579 | 463068 | 513 | 14976 |
| Nef+ 1 | Nef+NL4-3 | 21310653 | 23215 | 14947 | 382247 | 442 | 15311 |
| Nef+ 2 | Nef+NL4-3 | 23473640 | 21482 | 14127 | 509083 | 487 | 14912 |
| Nef- 1 | Nef-NL4-3 | 19433787 | 22525 | 14558 | 311271 | 403 | 14426 |
| Nef- 2 | Nef-NL4-3 | 23795137 | 22312 | 14575 | 426127 | 494 | 15088 |
| QVD 1 | BaL+QVD | 16262691 | 22626 | 13536 | 153463 | 338 | 11606 |
