# Supplementary material for: HIV-1 release requires Nef-induced caspase activation
Source: PLoS One. 2023 Feb 13;18(2):e0281087. doi: 10.1371/journal.pone.0281087 (PMC9925082; doi:10.1371/journal.pone.0281087)
Supplement: S1 Fig — (A) Enzymatic cleavage of a fluorescent peptide substrate ES003 (R&D systems, Inc) or a fluorescent CD62L peptide by recombinant ADAM10 (R&D systems, Inc.). Each enzymatic reaction consists of 400 ng of recombinant ADAM10 mixed with 2 μM ES003 (left panel) or 10 μM CD62L (right panel) peptides in the presence or absence of 1 μM specified inhibitor compounds in 100 μl reaction buffer using a 96-well microtiter plate. (B) FACS analyses on the percentage of CD62L+ (left panel) and CD4+ (right panel) T cells in p24+ (red), p24- (green) infected or uninfected (black) samples. Caspase activation correlated with the loss of CD62L and CD4 expressions. The data is representative of three independent experiments. Mann-Whitney nonparametric test *p < 0.05, **p < 0.01, ***p < 0.001. (C) Volcano plot of fold change in gene expression between HIV-1BAL infected and uninfected CD4 T lymphocytes by day 6 (from representative data in Fig 1D). Genes including members from IFI, IL, CCL, CCR, CXCR, and CDC groups involved in signaling and cell cycle regulation with significant changes in expression are highlighted in red (up-regulated) or blue (down-regulated), whereas genes involved in transcriptional regulation are highlighted in green. Among the most significantly up-regulated genes in infected cells are several interleukins and their receptors, such as IL-1β, IL-8, IL-18R and IL-23R. (PPTX) [file pone.0281087.s002.pptx]

## Slide 1
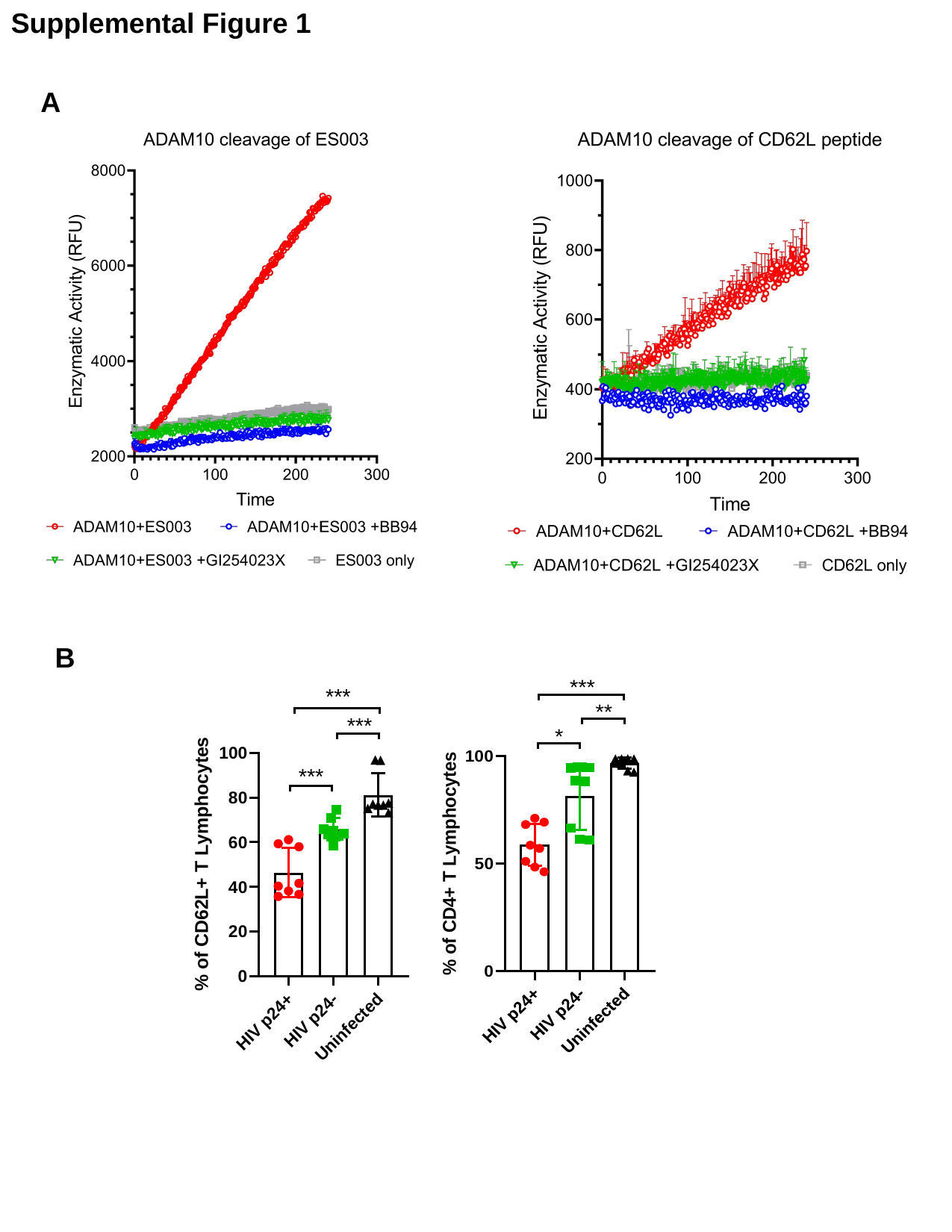

Supplemental Figure 1
A
B

## Slide 2
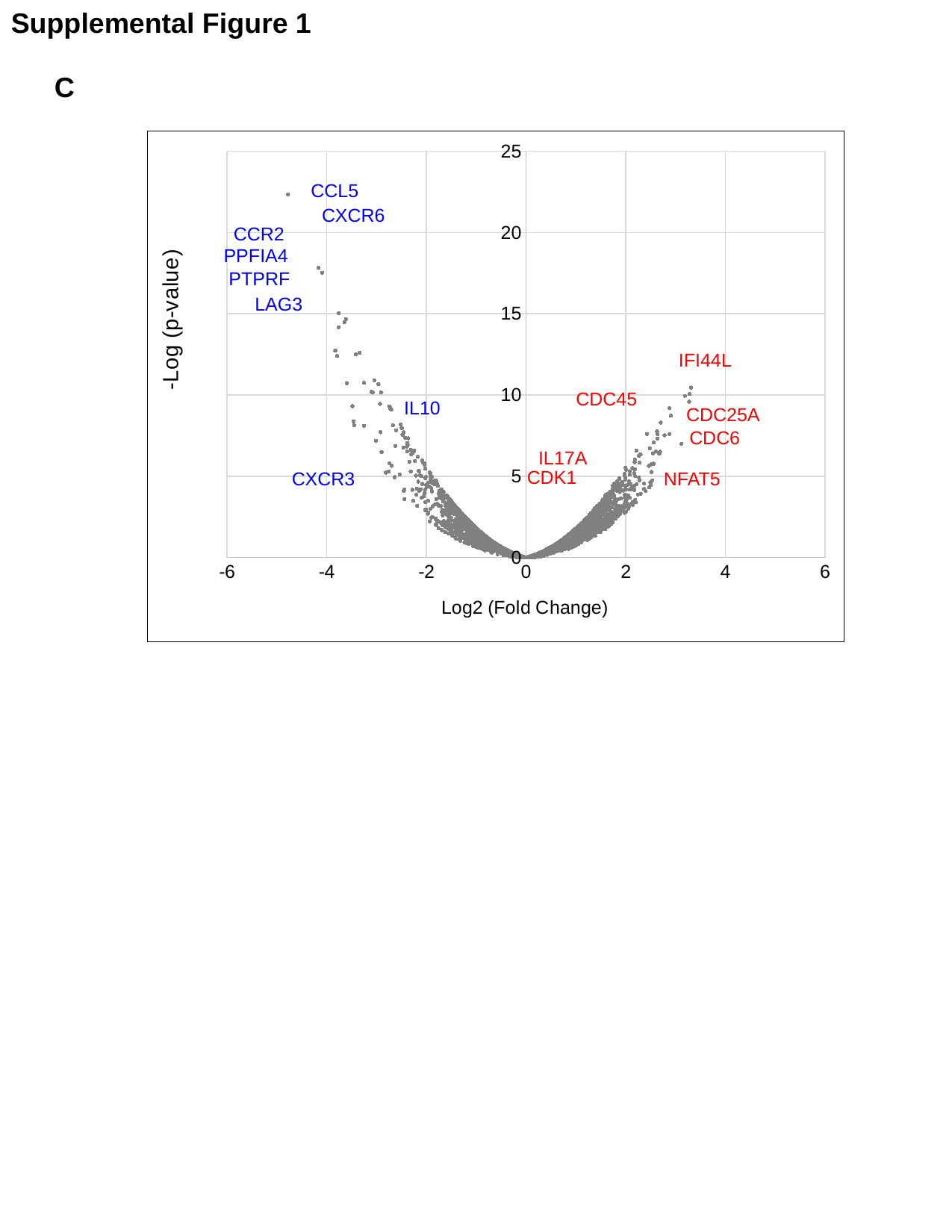

Supplemental Figure 1
C
### Chart
| Category | [-log10pvalue] | down | up | trxn/trsln |
|---|---|---|---|---|CCL5
CXCR6
CCR2
PPFIA4
PTPRF
LAG3
IFI44L
IL10
CDC45
CDC25A
CDC6
IL17A
CDK1
CXCR3
NFAT5
