# Supplementary material for: HIV-1 release requires Nef-induced caspase activation
Source: PLoS One. 2023 Feb 13;18(2):e0281087. doi: 10.1371/journal.pone.0281087 (PMC9925082; doi:10.1371/journal.pone.0281087)
Supplement: S2 Fig — Differential expressions of members of Interferon-inducible (A), TRIM (B), Chemokine receptors (CCR) and SERINC (C) family genes by RNA-Seq analyses on CD4 T cells enriched from day 6 in vitro HIV-1BAL infected PBMC from 3 donors. Several members of interferon-inducible genes such as IFI16 transcriptional activator and antiviral sensory responders IFI44 and IFIT (interferon-inducible transmembrane protein) are upregulated in the presence of the infection (A). Several antiviral response factors such as the tripartite motif family members (TRIM) including TRIM5, 22, and 56 are upregulated in response to HIV-1BAL infection in CD4 T cells from all 3 donors (B). HIV-1BAL infection upregulated the expressions of restriction factors SERINC5, SAMHD, and tetherin (BST2) but downregulated expressions of chemokine receptors (C). (D) RT-PCR probe of day 3 HIV-1BAL infected PBMC for the expressions of inflammatory (caspase 1), executioner (caspase 3), and initiator (caspase 8) classes of caspases. The results are displayed relative to that of beta-actin control. The statistics are calculated using student-test **p < 0.01, ***p < 0.001, ****p < 0.0001. (E) Representative of FACS plots from FLICA staining of individual activated caspases in primary lymphocytes on day 7 of post infection with HIV-1BAL. CD3+ cells treated with DMSO or QVD-OPH were gated on p24 capsid (top) before observing expression levels for each caspase. Samples treated with DMSO with defined populations of p24+ (red boxes) vs p24- (black boxes) were gated separately (left columns) while QVD-OPH and uninfected controls were gated on their total CD3+ populations (right columns). The results are representative of at least two experiments. (F) Activation of caspases in panel E grouped by initiator (caspases 2,8,9 10), executioner (caspases 3.6,7), and inflammatory (caspase 1) classes. *p < 0.05, ***p < 0.0005. (PPTX) [file pone.0281087.s003.pptx]

## Slide 1
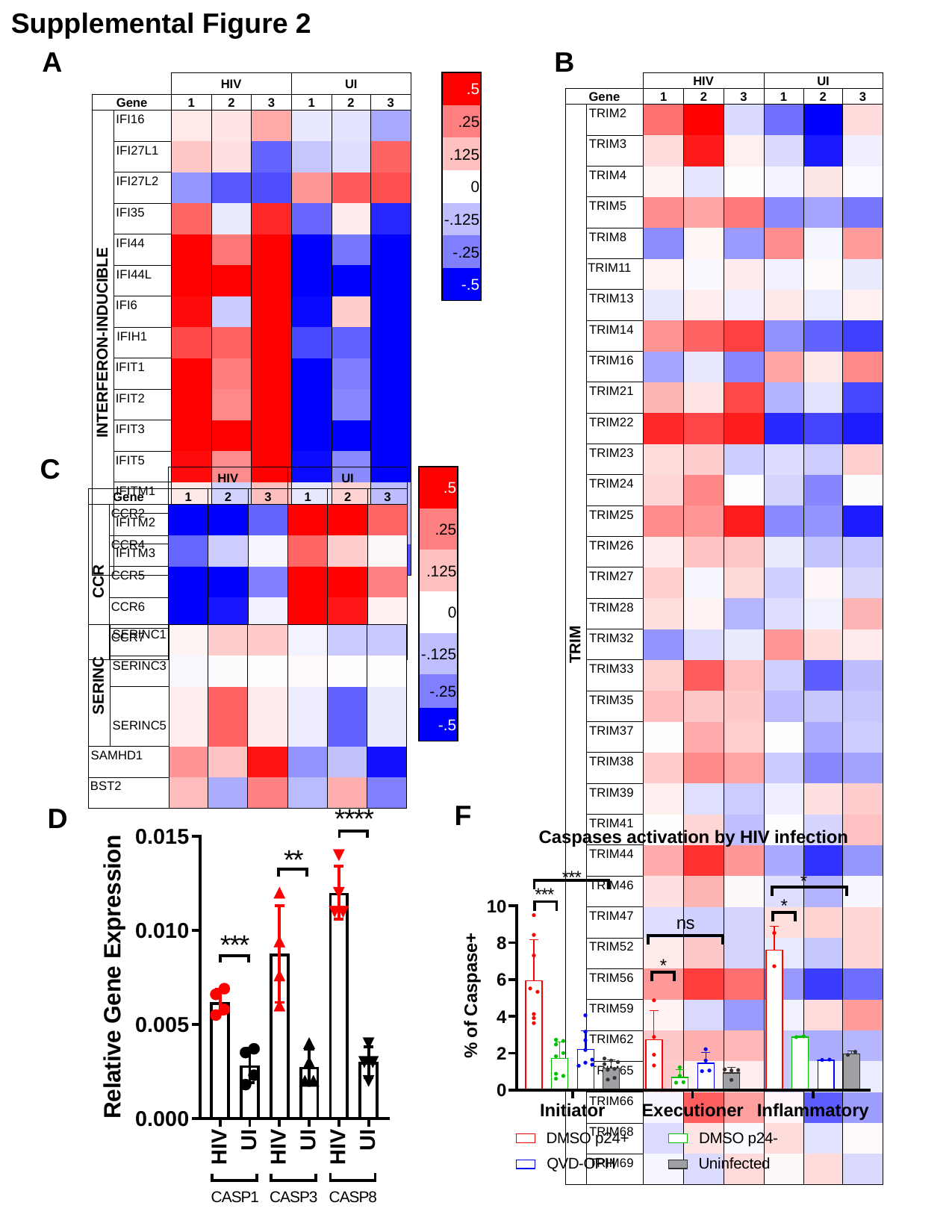

Supplemental Figure 2
B
A
| | | | HIV | | | UI | | |
| --- | --- | --- | --- | --- | --- | --- | --- | --- |
| Gene | | | 1 | 2 | 3 | 1 | 2 | 3 |
| INTERFERON-INDUCIBLE | IFI16 | IFI16 | | | | | | |
| | IFI27L1 | IFI27L1 | | | | | | |
| | IFI27L2 | IFI27L2 | | | | | | |
| | IFI35 | IFI35 | | | | | | |
| | IFI44 | IFI44 | | | | | | |
| | IFI44L | IFI44L | | | | | | |
| | IFI6 | IFI6 | | | | | | |
| | IFIH1 | IFIH1 | | | | | | |
| | IFIT1 | IFIT1 | | | | | | |
| | IFIT2 | IFIT2 | | | | | | |
| | IFIT3 | IFIT3 | | | | | | |
| | IFIT5 | IFIT5 | | | | | | |
| | IFITM1 | IFITM1 | | | | | | |
| | IFITM2 | IFITM2 | | | | | | |
| | IFITM3 | IFITM3 | | | | | | |
| .5 |
| --- |
| .25 |
| .125 |
| 0 |
| -.125 |
| -.25 |
| -.5 |
| | | | HIV | | | UI | | |
| --- | --- | --- | --- | --- | --- | --- | --- | --- |
| Gene | | | 1 | 2 | 3 | 1 | 2 | 3 |
| TRIM | TRIM2 | TRIM2 | | | | | | |
| | TRIM3 | TRIM3 | | | | | | |
| | TRIM4 | TRIM4 | | | | | | |
| | TRIM5 | TRIM5 | | | | | | |
| | TRIM8 | TRIM8 | | | | | | |
| | TRIM11 | TRIM11 | | | | | | |
| | TRIM13 | TRIM13 | | | | | | |
| | TRIM14 | TRIM14 | | | | | | |
| | TRIM16 | TRIM16 | | | | | | |
| | TRIM21 | TRIM21 | | | | | | |
| | TRIM22 | TRIM22 | | | | | | |
| | TRIM23 | TRIM23 | | | | | | |
| | TRIM24 | TRIM24 | | | | | | |
| | TRIM25 | TRIM25 | | | | | | |
| | TRIM26 | TRIM26 | | | | | | |
| | TRIM27 | TRIM27 | | | | | | |
| | TRIM28 | TRIM28 | | | | | | |
| | TRIM32 | TRIM32 | | | | | | |
| | TRIM33 | TRIM33 | | | | | | |
| | TRIM35 | TRIM35 | | | | | | |
| | TRIM37 | TRIM37 | | | | | | |
| | TRIM38 | TRIM38 | | | | | | |
| | TRIM39 | TRIM39 | | | | | | |
| | TRIM41 | TRIM41 | | | | | | |
| | TRIM44 | TRIM44 | | | | | | |
| | TRIM46 | TRIM46 | | | | | | |
| | TRIM47 | TRIM47 | | | | | | |
| | TRIM52 | TRIM52 | | | | | | |
| | TRIM56 | TRIM56 | | | | | | |
| | TRIM59 | TRIM59 | | | | | | |
| | TRIM62 | TRIM62 | | | | | | |
| | TRIM65 | TRIM65 | | | | | | |
| | TRIM66 | TRIM66 | | | | | | |
| | TRIM68 | TRIM68 | | | | | | |
| | TRIM69 | TRIM69 | | | | | | |
C
| .5 |
| --- |
| .25 |
| .125 |
| 0 |
| -.125 |
| -.25 |
| -.5 |
| | | | HIV | | | UI | | |
| --- | --- | --- | --- | --- | --- | --- | --- | --- |
| Gene | | | 1 | 2 | 3 | 1 | 2 | 3 |
| CCR | CCR2 | CCR2 | | | | | | |
| | CCR4 | CCR4 | | | | | | |
| | CCR5 | CCR5 | | | | | | |
| | CCR6 | CCR6 | | | | | | |
| | CCR7 | CCR7 | | | | | | |
d
| SERINC | SERINC1 | | | | | | |
| --- | --- | --- | --- | --- | --- | --- | --- |
| | SERINC3 | | | | | | |
| | SERINC5 | | | | | | |
| SAMHD1 | | | | | | | |
| BST2 | | | | | | | |
F
D

## Slide 2
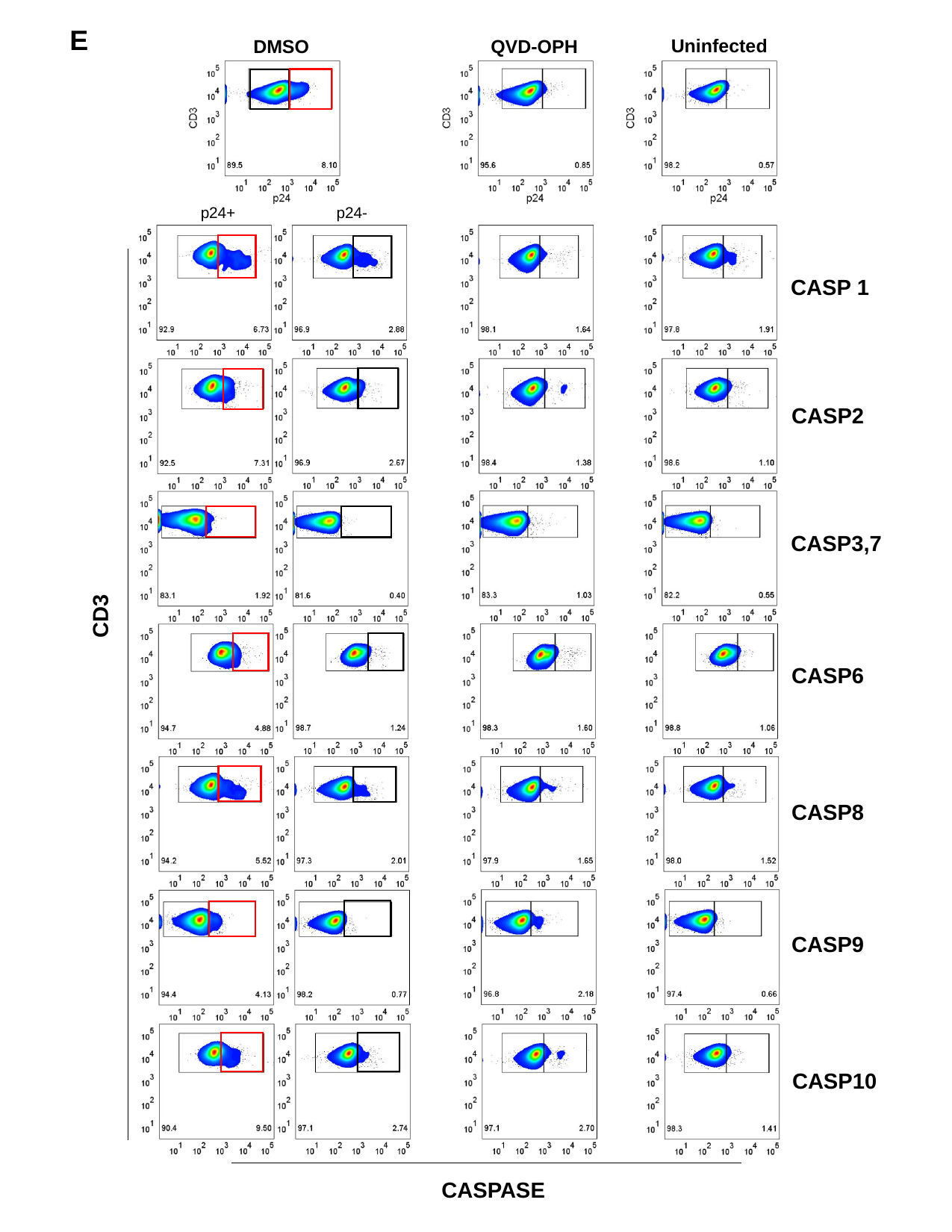

E
Uninfected
QVD-OPH
DMSO
p24-
p24+
CASP 1
CASP2
CASP3,7
CD3
CASP6
CASP8
CASP9
CASP10
CASPASE
