# Supplementary figures and images for: HIV-1 release requires Nef-induced caspase activation
Source: PLoS One. 2023 Feb 13;18(2):e0281087. doi: 10.1371/journal.pone.0281087 (PMC9925082; doi:10.1371/journal.pone.0281087)

## Slide 1
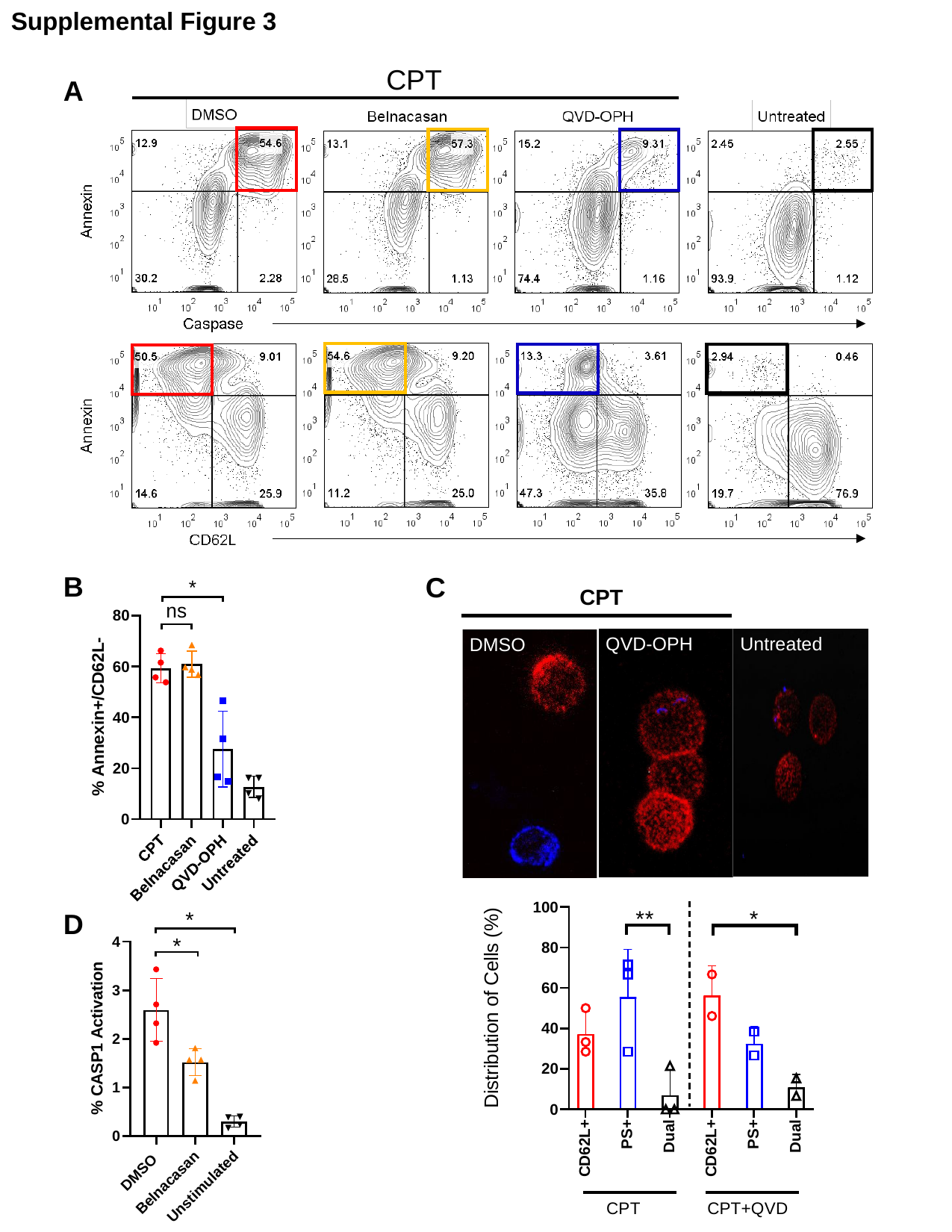

Supplemental Figure 3
CPT
A
B
C
CPT
Untreated
QVD-OPH
DMSO
D

## Slide 2
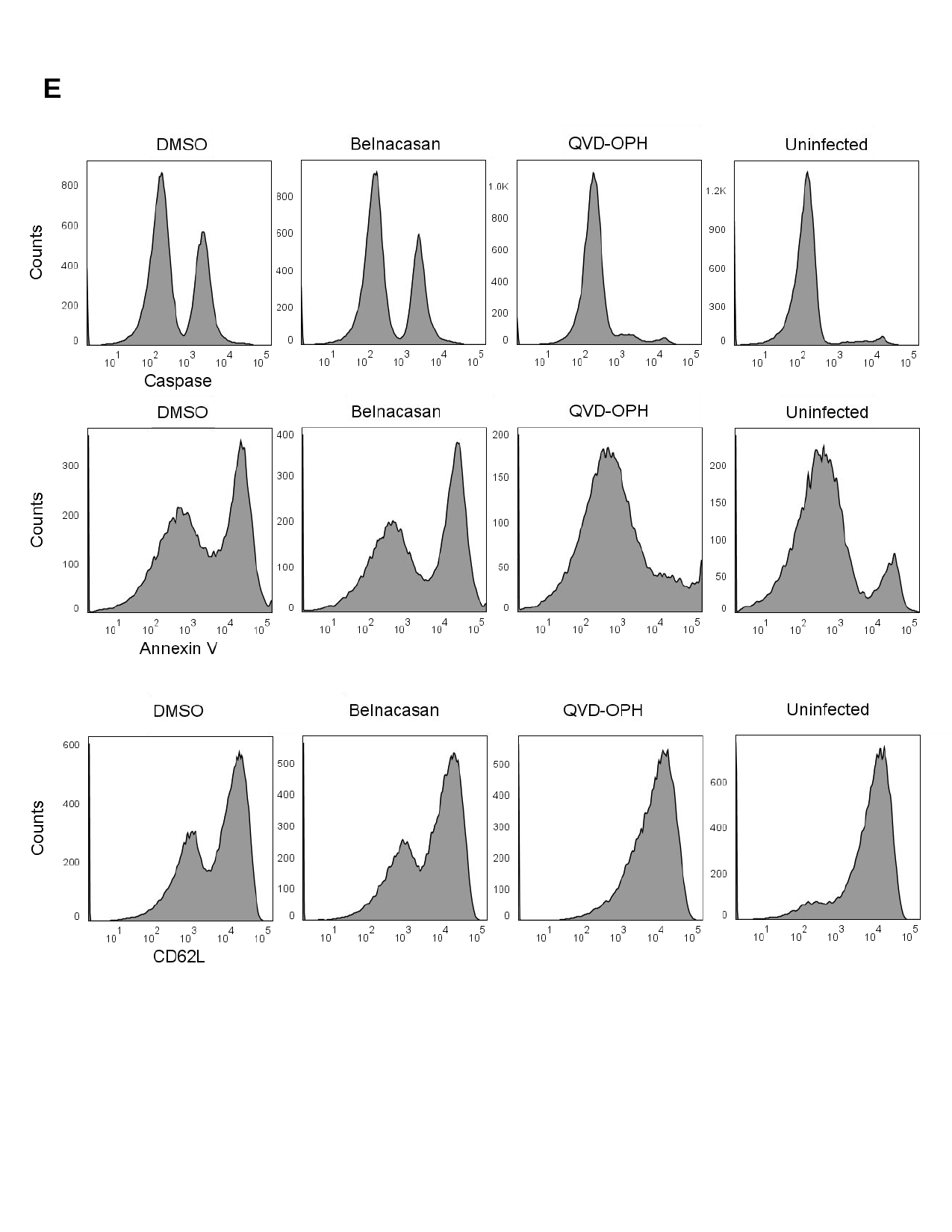

E

Supplement: S3 Fig — (A) Representative FACS contour plots of primary CD4+ T cells treated with 10 μM CPT in the presence or absence of Belnacasan (orange), QVD-OPH (blue) or DMSO controls. Annexin V+/caspase+ and annexin V+/CD62L- populations are highlighted in colored gates. QVD-OPH but not Belnacasan significantly reduced annexin V+/caspase+ and annexin V+/CD62L- populations. (B) Statistical quantification of annexin V+/CD62L- populations within specified gates in panel A from two independent experiments. Mann-Whitney nonparametric test *p < 0.05. (C) Confocal microscopy representative of two independent experiments of CPT treated and untreated primary CD4+ T cells stained with annexin V (blue) and anti-CD62L (red). The majority of cells were stained with either anti-CD62L or Annexin V but not both. The lower bar diagram shows percentage of cells labeled with CD62L (red bars), PS (blue bars), or both (dual, black bars) in confocal images under CPT treatment in the presence and absence of QVD-OPH. The statistical analysis was done using 2-way ANOVA with Tukey’s method *p < 0.05, **p < 0.01. (D) Bar diagram showing the inhibition of CASP1 activation by 50 μM Belnacasan (orange) versus DMSO control (red). Mann-Whitney nonparametric student t test *p < 0.05. (E) Histograms for the activation of caspases, annexin V staining and expression of CD62L corresponding to the FACS analyses presented in Fig 2A and 2B. (PPTX) [file pone.0281087.s004.pptx]

## Slide 1
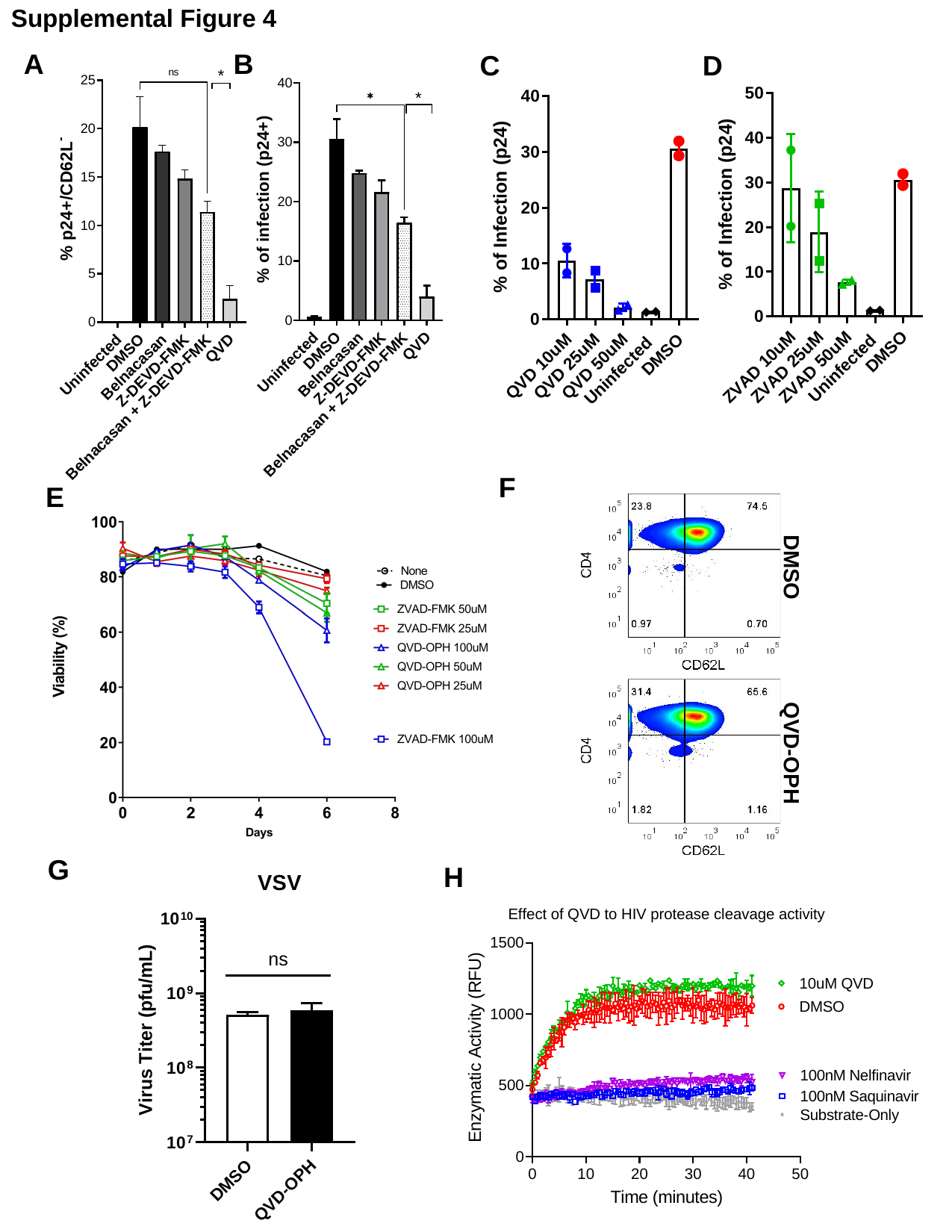

Supplemental Figure 4
A
B
C
D
F
E
DMSO
QVD-OPH
G
H

## Slide 2
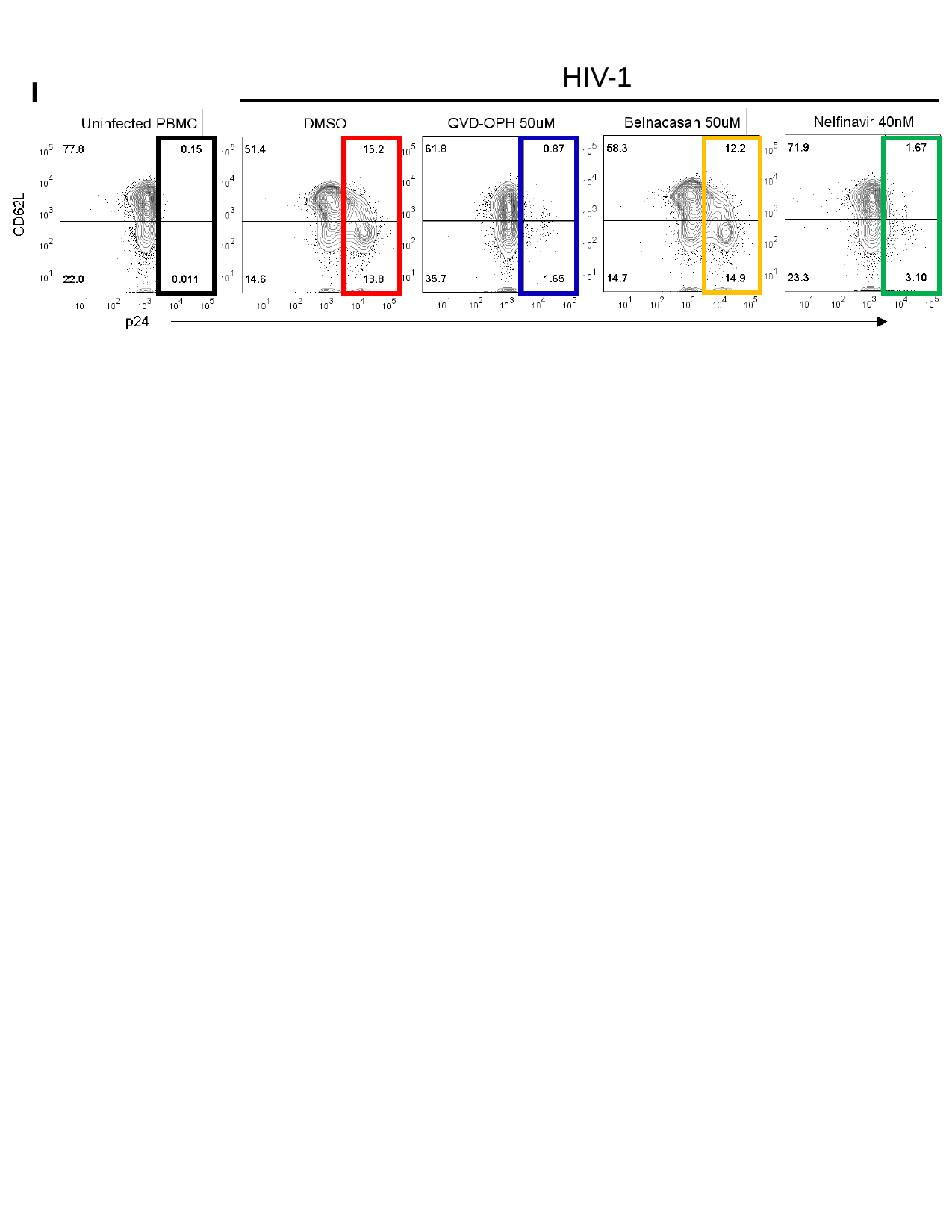

HIV-1
I

Supplement: S4 Fig — (A-B) Effect of combination of two caspase inhibitors to HIV-1 infection. Stimulated, CD8-depleted PBMC were infected with HIV-1BAL in the presence of caspase inhibitors Belnacasan (caspase 1 and 4), Z-DEVD-FMK (caspase 3,6,7 and 10), the combination of the two compounds, QVD-OPH or control DMSO. All compounds were used at 50μM concentration in duplicates. The infections were analyzed on day 7 of post infection by FACS for the percentage of infected cells that lost CD62L (A) and for the infection level (B). (C-D) Dose-dependent inhibition of HIV-1BAL infection by QVD-OPH (C) or ZVAD-FMK (D). (E) Viability of PBMC treated with titration concentrations of ZVAD-FMK and QVD-OPH in triplicates. (F) The expressions of CD4 and CD62L on anti-CD3 stimulated PBMC on Day 7 in the absence and presence of 50 μM QVD-OPH. Treatment of QVD-OPH did not affect the expression of CD4 and CD62L. (G) Effect of caspase inhibition to VSV infection. PBMC infected with replication-competent vesicular stomatitis virus (VSV Indiana strain) in the presence of caspase inhibitor QVD-OPH or control DMSO. Data from two independent experiments. (H) Effect of caspase inhibitor QVD-OPH to HIV protease enzymatic cleavage of a fluorescent Gag peptide. The cleavage reaction was carried out in the presence of 100nM viral protease inhibitor, Saquinavir, Nelfinavir, 10μM caspase inhibitor QVD-OPH or control DMSO. (I) Representative of one out of four infection experiments used for immunoblots (Fig 5I–5K). CD8-depleted PBMC’s were infected with HIV-1BAL in the presence of DMSO (red), QVD-OPH (blue), Belnacasan (orange), or Nelfinavir (green), and analyzed for total infections (p24+) in CD3+ populations. (PPTX) [file pone.0281087.s005.pptx]

Loading: Supernatant UI, Nel, QVD, DMSO, Lysate: UI, Nel, QVD, DMSO, Marker

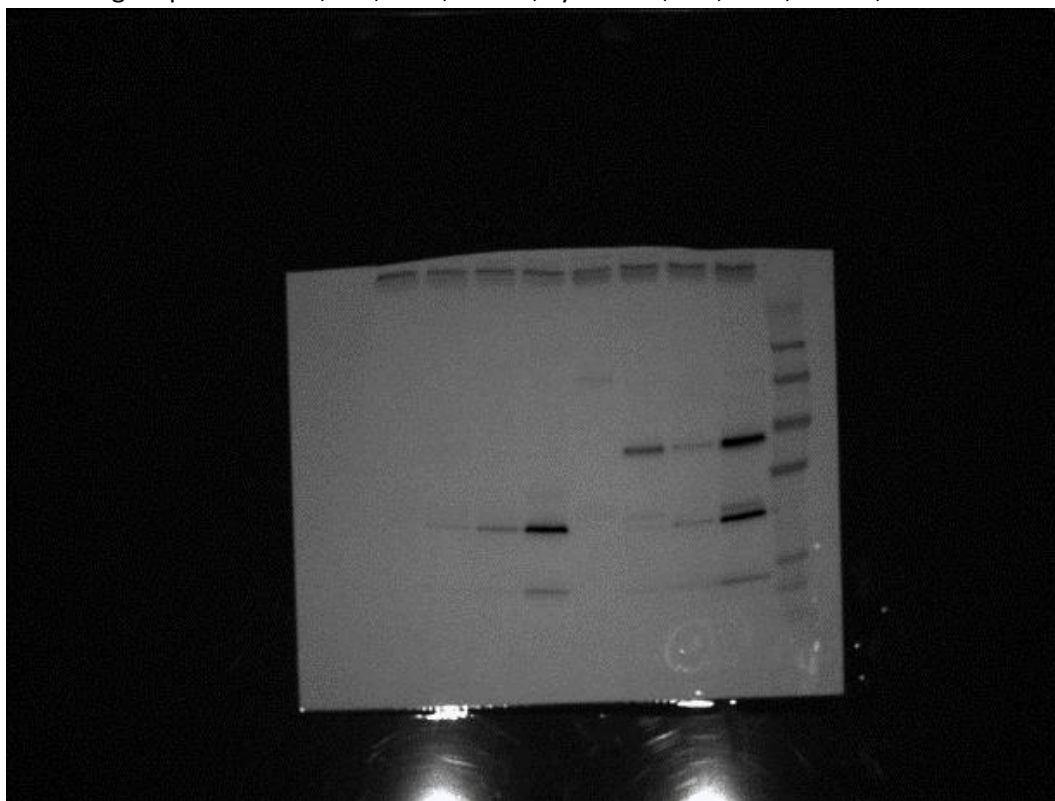

Beta-Actin

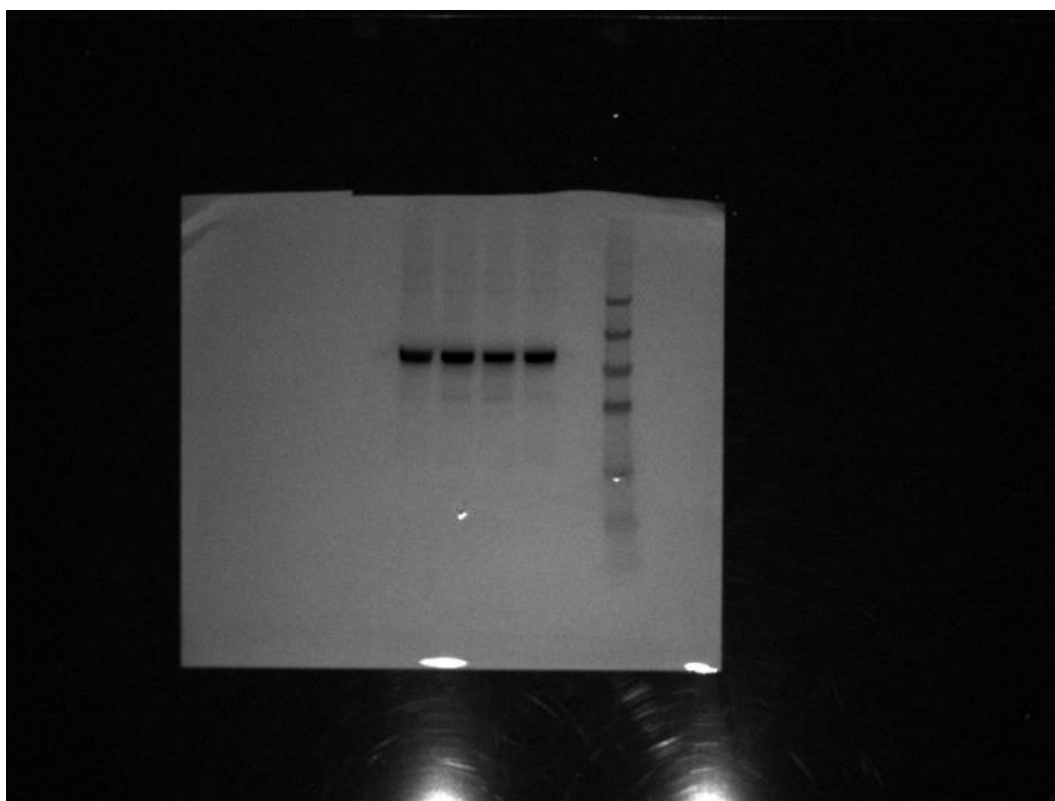

Supplement: S1 Raw images — (PDF) [file pone.0281087.s007.pdf]
