# Supplementary material for: HIV-1 release requires Nef-induced caspase activation
Source: PLoS One. 2023 Feb 13;18(2):e0281087. doi: 10.1371/journal.pone.0281087 (PMC9925082; doi:10.1371/journal.pone.0281087)
Supplement: S5 Fig — (A) Representative transmission-EM image of PBMC’s infected with HIV-1BAL in the presence of Belnacasan which consistently failed to suppress infection in all assays. Scale bar = 200 nm. (B) Fraction of virions associated with gold-conjugated anti-CD62L in the presence of QVD-OPH or DMSO. The percentage is tallied against the total number of virions observed in individual TEM images. Student t-test *p < 0.05. (C-D) Population of T cells stained double positive for caspase and annexin V (C) or double negative for CD4 and CD62L in Nef+, Nef- HIVNL4-3 infected PBMC or uninfected samples (D). The FACS analyses were performed on CD3 positive T cells. Student-test **p < 0.01. (E). Nef sufficient (white bars) and deficient (ΔNef, grey bars) virus infection of CEM#2 cells in the absence and presence of 50μM QVD. (F) RNA-Seq-based differential gene expression analyses on primary CD4 T cells infected with Nef+ or Nef- HIV-1NL4-3 from 2 donors for genes involved in TLR or IL-2 signaling pathways. (G) Protein-interaction network connecting various apoptotic pathways as depicted by Cytoscape (http://cytoscape.org) and String-Db (http://string-db.org). The shared components among the pathways are highlighted in red box. (PPTX) [file pone.0281087.s006.pptx]

## Slide 1
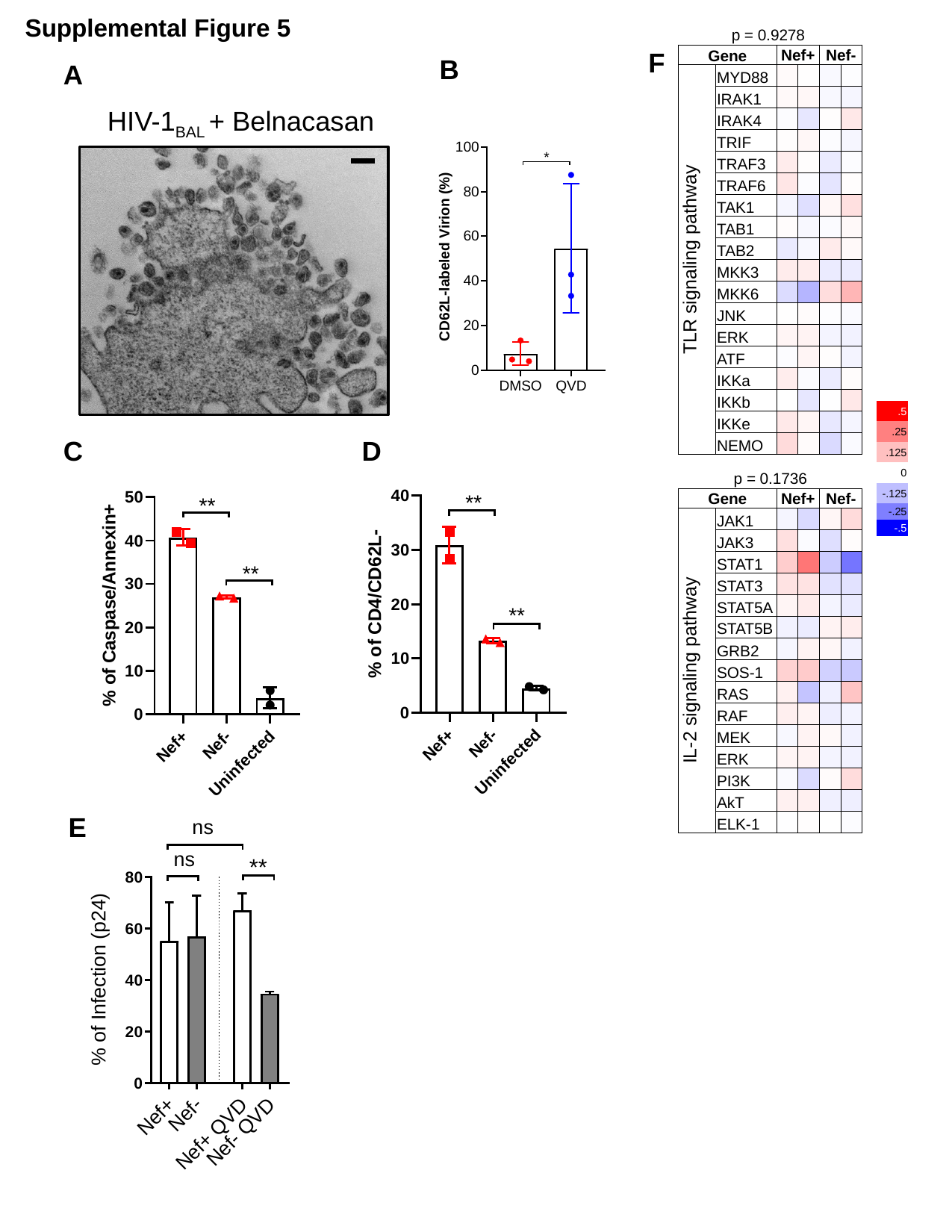

Supplemental Figure 5
| p = 0.9278 | | | | | |
| --- | --- | --- | --- | --- | --- |
| Gene | | Nef+ | | Nef- | |
| TLR signaling pathway | MYD88 | | | | |
| | IRAK1 | | | | |
| | IRAK4 | | | | |
| | TRIF | | | | |
| | TRAF3 | | | | |
| | TRAF6 | | | | |
| | TAK1 | | | | |
| | TAB1 | | | | |
| | TAB2 | | | | |
| | MKK3 | | | | |
| | MKK6 | | | | |
| | JNK | | | | |
| | ERK | | | | |
| | ATF | | | | |
| | IKKa | | | | |
| | IKKb | | | | |
| | IKKe | | | | |
| | NEMO | | | | |
F
B
A
HIV-1BAL + Belnacasan
| .5 |
| --- |
| .25 |
| .125 |
| 0 |
| -.125 |
| -.25 |
| -.5 |
D
C
| p = 0.1736 | | | | | |
| --- | --- | --- | --- | --- | --- |
| Gene | | Nef+ | | Nef- | |
| IL-2 signaling pathway | JAK1 | | | | |
| | JAK3 | | | | |
| | STAT1 | | | | |
| | STAT3 | | | | |
| | STAT5A | | | | |
| | STAT5B | | | | |
| | GRB2 | | | | |
| | SOS-1 | | | | |
| | RAS | | | | |
| | RAF | | | | |
| | MEK | | | | |
| | ERK | | | | |
| | PI3K | | | | |
| | AkT | | | | |
| | ELK-1 | | | | |
E

## Slide 2
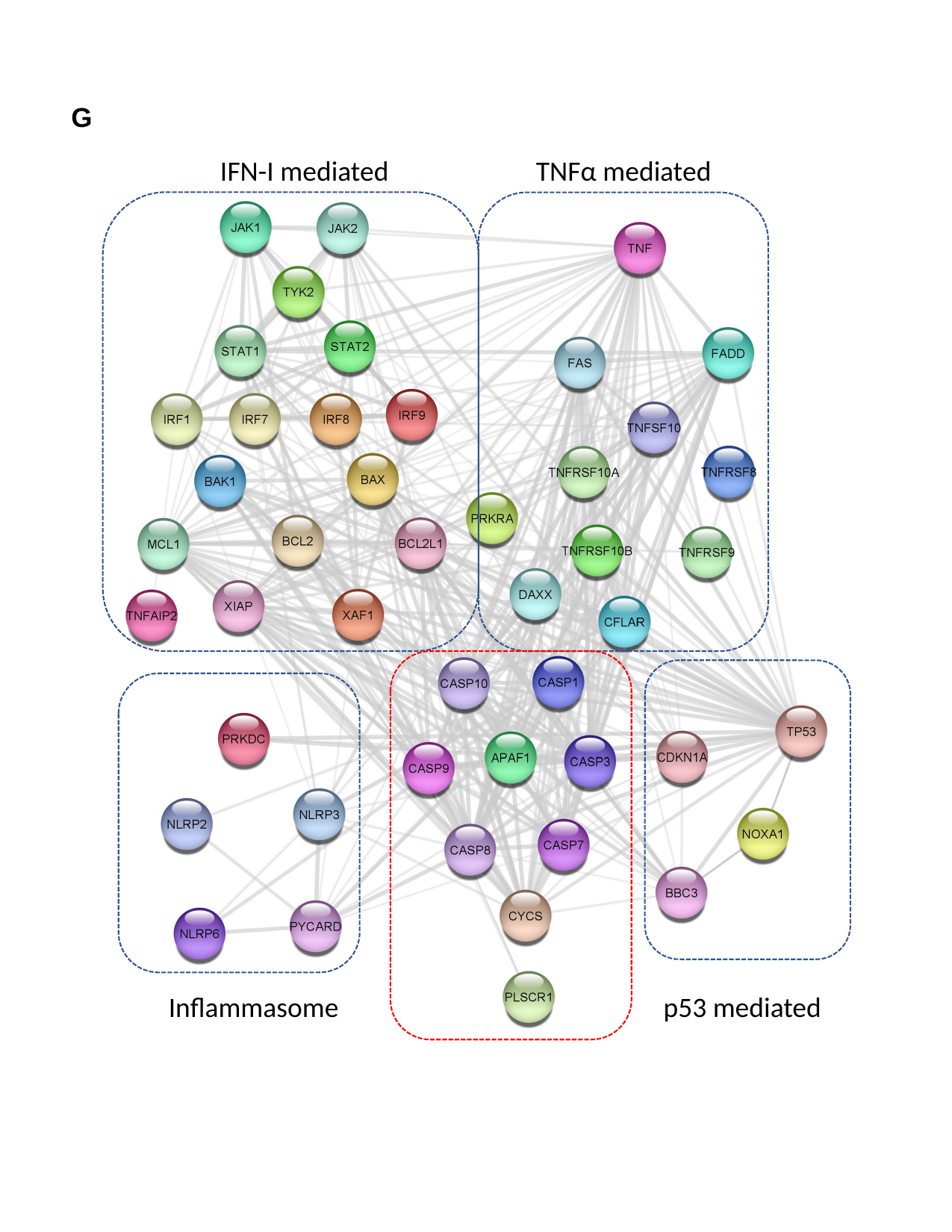

G
IFN-I mediated
TNFα mediated
Inflammasome
p53 mediated
