# Supplementary material for: HIV-1 release requires Nef-induced caspase activation
Source: PLoS One. 2023 Feb 13;18(2):e0281087. doi: 10.1371/journal.pone.0281087 (PMC9925082; doi:10.1371/journal.pone.0281087)
Supplement: Attachment — Submitted filename: rebuttal.docx [file pone.0281087.s008.docx]

Rebuttal to the reviewer’s comments.

Reviewer 1’s comments reflect several apparent misunderstandings to our manuscript which resulted in suggestions that are conceptually incorrect. For example, the reviewer asked us to show “caspase cleavage of CD62L”. Caspase does not cleave CD62L, and the suggestion is a misunderstanding to our manuscript, which shows caspase activation of ADAM sheddases, which in turn cleaves CD62L. These misunderstandings presumably guided the reviewer’s unfair review. We only realized the incorrect assessment to our work from the reviewer’s latest comments to our revision. In the previous review, the reviewer did not clearly state his/her view and we did not realize the presence of these misunderstandings. In the review to the final revision, the reviewer further clarified those previous comments. These clarifications in the latest comments from reviewer 1 made us to realize that they are based on conceptual misunderstanding from the reviewer. We highlight the misunderstandings from reviewer 1 below, which form bulk of the criticism from the reviewer and provide our explanation. In total, reviewer 1 raised 6 comments to our revised manuscript. Three of them (comments 1,2 &5) represent series misunderstanding to our manuscript. Comment 3 is technically not feasible and lack sound scientific reasoning for performing it. Comments 4 and 6 were performed with experiments fulfilling the criticism, however, was not acknowledged by the reviewer, suggesting a bias in the reviewer.

As the reviewer did not clearly state his/her misunderstanding in earlier reviews, we did not realize that there are series conceptual misunderstandings, which we presume form the basis of the reviewer’s claim that we failed to address his/her concern. Since the reviewer’s comments are based on some conceptual misunderstanding, we respectfully request the editor to discount the reviewer’s comments.

Reviewer 2’s criticism is primarily on writing of the figure legend and clarification of a statement. We have implemented the changes accordingly in the submission to PLOS One.

The following paragraphs list the detailed responses to each comment. The reviewers’ comments highlighted in italic.

***Comment 1 from Reviewer #1****: This revised manuscript by Segura et al discusses some of the reviewer critiques from the initial submission, but importantly does not experimentally address many of the major concerns. Initial reviews requested additional experimental data, and critical experiments were not performed, and so key conclusions are not fully supported by experimental data.*

*Specifically in the revised manuscript,*

*-There is no mechanism between viral release and caspase activation; only an association which has been previously reported many times.*

**Response**: The reviewer misunderstood the main theme of our manuscript. The association between HIV-1 viral release and caspase activation has never been reported before. What has been reported before was “HIV infection activates caspases.” However, as we stated in the introduction that the current paradigm for the viral induced caspase activation is centered on host immune and antiviral response. Namely, caspase activation is part of cellular antiviral response to kill infected T cells. This has been the guiding principle in current therapeutic approach to develop compounds to enhance apoptotic death of infected T cells. What we reported here is the opposite of the current paradigm. We showed here that the viral-induced caspases activation is required for viral release. We did not need to show HIV infection activates caspases—this has been well established as the reviewer stated. This part of data was necessary in our manuscript to connect to the viral release part. Otherwise, the manuscript may be subject to other criticisms, such as not knowing the activation status of the caspases, etc. We provided solid evidence to show that caspase activation resulted in viral release—a novel observation that has not been published before. This new understanding has profound implication to the current HIV therapeutic approach. It suggests an opposite approach to inhibit caspase activation and thus to inhibit viral release rather than the current strategy of enhancing procaspases expression with compounds, such as flurbiprofen (a caspase 8 inducer). Based on our findings, caspases expression inducer compounds may stimulate HIV viral release and enhance viral transmission, thus should be avoided.

***Comment 2 from Reviewer #1****: -Statements such as "viral infection induced caspase activation...directly contributes to Cd62L shedding" remain unsupported (the only data support an association, and experiments linking the two have not been performed eg caspase cleavage of CD62L)*

**Response**: This is a conceptual misunderstanding to our manuscript. We do not think caspase cleaves CD62L. We have stated clearly in the introduction that CD62L was shed by ADAM metalloproteinases and showed in Figure 1A of CD62L cleavage by membrane-associated metalloproteinases on infected T cells. We have never stated nor implied caspases cleavage of CD62L. Since ADAM activation is dependent on caspase-mediated PS-flipping, as published by others, we investigated in this manuscript the contribution of caspase to CD62L shedding and viral release through ADAM activation and PS-flipping. We showed in Figure 2 that HIV infection induced PS-flipping and caspase activation. Apparently, the reviewer regard “caspase cleavage of CD62L” as the direct supporting evidence. This is a clearly incorrect statement from the reviewer to our manuscript. Conceptually, caspases are intracellular enzymes that would not be accessible to the shedding of CD62L, whose cleavage site is located at extracellular membrane proximal region. Thus, it is inappropriate to investigate caspase cleavage of CD62L.

***Comment 3 from Reviewer #1****:- the results using Caspase inhibitors are over interpreted, under the premise that they are highly specific; conclusions should be complemented by orthogonal experiments such as caspase knockdown*

**Response**: The requested experiment is not technically feasible to our knowledge for primary PBMC/CD4 T cells. Previous publications by others regarding to identification of HIV restriction factors, such as SERINC5, the knockdown experiments were performed in cell lines. While caspase knockdown is feasible in cell lines, HIV release in cell lines, such as CEM, are independent on caspases. As we showed in Supplemental figure 5G, QVD did not inhibit Nef+ HIV infection of CEM cells. In addition, we observed a significant redundancy in caspases function to HIV release as we showed that single-caspase specific compounds were not effective in inhibiting HIV infection (Figure 3). Therefore, single caspase knockdown will likely be inconclusive. Caspase activation for viral release is likely a primary cell phenomenon as primary PBMC express high level of CD62L but does not shed CD62L without activation. The reason caspase is not required in cell lines is not entirely clear but may be related to their lower level of CD62L expression and the presence of constitutively shedding reported in CEM cells.

***Comment 4 from Reviewer #1****:- the authors did not control for the effects of Nef deletions and mutants on viral fitness*

**Response**: As nef is known required for HIV transmission in primary infections but dispensable in the viral propagation in cell lines, namely viral replication is not affected in cell lines. To control for nef’s effect in viral replication and release, we further performed nef+/- experiments in CEM cells in which there is no significant difference in viral infection between nef+ and nef- viruses (Supplemental Figure 5G), suggesting no replication and other fitness deficiencies associated with nef in the cell line. However, the nef- HIV infection of CEM was impaired compared to that of nef+ HIV in the presence of caspase inhibitor QVD (Supplemental Figure 5G), suggesting nef counters QVD inhibition of the caspase.

***Comment 5 from Reviewer #1****:*- *there is no mechanistic link, or direct evidence of interaction between Nef and CD62L cleavage*

**Response**: This is another comment likely related to the reviewer’s misunderstanding to our manuscript. The reviewer asked for evidence to show direct interaction between Nef and CD62L cleavage. In principle, Nef does not need to interact with CD62L nor ADAM metalloproteinases to induce L-selectin cleavage. All nef needs to do is to stimulate the pathway leading to ADAM activation, or stimulate caspases which lead to ADAM activation. We do not think Nef directly interact with ADAMs as ADAMs are cell surface enzymes. Nef’s effect is likely affecting upstream signaling events leading to caspase activation. It would not be direct interaction nor need to be such with CD62L cleavage enzymes. Thus, the reviewer’s comment represent a conceptual misunderstanding to our manuscript.

***Comment 6 from Reviewer #1****:*- *the conclusions that caspase inhibition prevent release of HIV from reservoirs is not controlled for cellular activation (which caspase inhibitors also antagonize)*

**Response**: We have addressed the “cellular activation” concern in our revised manuscript. In the revised manuscript, we performed the viral release assay without using CD3 stimulation. The viral release results using non-stimulated PBMC from three HIV patients are included in Figure 7C. While the total number of released viruses are reduced in the absence of CD3 stimulation, QVD-OPH suppressed HIV release in all three cases. We are puzzled by the reviewer’s current statement as we performed exactly the experiment to remove the involvement of CD3 stimulation in revised Figure 7C.

***Reviewer #2 comment 1:*** *In response to my previous concerns, the authors have replaced Fig 4F with a more convincing western blot, together with quantification, which now show convincingly that nelfinavir but not QVD reduced Gag cleavage.*

*However, many of my other concerns have not been addressed. In particular,*

*- “figure legends are minimalistic and it’s almost impossible to understand what was tested, at which concentration or time point, without going through the entire M&M section. For instance, Fig 4A does not explain how viral entry was measured and I could not find this anywhere in the manuscript.” Authors responded that they “have tried to clarify the figures”, however I fail to see how they achieved this since I did not find clarification to Fig 4A anywhere in the revised manuscript. In fact, none of the Fig legends appear to have been edited, the only changes are for the new panels.*

Response: Figure legends have been expanded and revised accordingly.

***Reviewer 2 comment 2:*** - “*authors are not looking at infection but viral spread in a 6-day culture… authors show that caspase inhibition does not inhibit entry (line 230, Fig 4A) so they are not inhibiting infection per se.” Authors responded that they “use infection in this manuscript to include viral entry, replication, spread and cell-cell transmission. It encompasses more than viral entry.” I did not find this clarification in the manuscript and see no edits to state that experiments measure viral spread or release or another step.*

Response: A statement is added to the methods section: “HIV infection here includes viral entry, replication, spread and cell-cell transmission in replication-competent viruses. It reflects viral entry in pseudovirus experiments.”
