# Supplementary material for: HIV-1 release requires Nef-induced caspase activation
Source: PLoS One. 2023 Feb 13;18(2):e0281087. doi: 10.1371/journal.pone.0281087 (PMC9925082; doi:10.1371/journal.pone.0281087)
Supplement: Attachment — Submitted filename: Response to reviewers comments-11282022.docx [file pone.0281087.s009.docx]

Response to reviewers comments:

We thank both reviewers for their in-depth review of our manuscript and for their constructive comments. We have provided further data and thoroughly revised both text and figure legends according to the reviewer’s suggestions. In brief, we added control enzymatic cleavage assays on CD62L peptide as the new Supplemental Figure 1 A to support the specificity of infected supernatant cleavage reported in Figure 1A. We included an ELISA measurement of shed CD62L in infected supernatant in Figure 1A to support HIV infection induced CD62L shedding. Histograms of caspases and annexin V associated with Figure 2A-B are included in Supplemental Figure 3E. Similar to the FACS contour plots presented in Figure 2A-B, these histograms also show the activation of caspases, down-regulation of CD62L, and the staining of annexin V upon HIV-1 infections, and they are inhibited by QVD-OPH but not Blenacasan. We also performed combination inhibitor assay, in which HIV-1 infection was treated with a combination of two different caspase inhibitors to address if combining two caspase inhibitors are sufficient to suppress the viral infection. The combined treatment resulted in marginal better inhibition than the single inhibitors, but still less than the pan-caspase inhibitor. This data is included as Supplemental Figure 4A-B. We also updated Supplemental Table 1 to include RNAseq data on Nef+ and Nef- HIV infections of CD4 T cells from two donors. The previous manuscript had RNAseq data from both donors but the supplemental table only included data from one of the two donors. In response to the reviewers comments on original manuscript being densely packed with data and figures making it less comprehensive, we extensively revised the text and re-wrote all figure legends. We moved IP-westernblot data on gag peptides from original Figure 4 to Figure 5 and removed Supplemental Figure 6. The following paragraphs are point-to-point response to the reviewers’ comments with the comments highlighted in italics.

*Reviewer #1: Efficient release of progeny virions from infected cells is the last crucial step in the viral replication cycle. Thus, successful pathogens like HIV evolved intricate strategies that allow their particles to efficiently detach from the cellular surface. CD62L, an L-selectin, was previously implicated in initial binding of HIV virions to cell surfaces, conversely the presence of CD62L also prevents efficient shedding of particles.

In this manuscript, Segura and co-workers suggest that the HIV accessory protein Nef induces caspase activation and subsequent CD62L shedding supporting viral release of HIV-1. The experiments indicate that in HIV-1 infected CD4+ T cells caspase activity/apoptosis is enhanced (like previously shown e.g. Rasola et al, JI, 2001; Doitsh et al, Nature, 2014 and many others). Upon using pan-caspase inhibitors QVD-OPH infection rate were reduced. However, key experiments are still required to support the major claims of this manuscript.
There are many mechanistic ‘jumps’ and gaps in the data that result in overinterpretation in the final model. Can the authors show that CD62L is actually cleaved/shed in the process (e.g. by supernatant blots)? How do the authors propose that Caspases are upregulated and then (which is required for many caspases) activated by Nef? It was previously shown that Vpu and Nef downregulate CD62L surface expression independent of Metalloproteases (Vassena et al, JVI, 2015). Is the model proposed by the authors via caspases, dependent or independent of MMPs. This has to be addressed experimentally (e.g. MMP inhibition) and clarified whether similar mechanisms are at play (sequestration of CD62L).*

Response: The shedding of CD62L during HIV-1 infection has been demonstrated using ELISA in our previous publication (Kononchik et al. Nat Commu 2018). The mode we proposed for CD62L shedding is dependent on ADAM family of MMPs (Kononchik et al. Nat Commu 2018). To further address the reviewer’s comment in this manuscript to support the cleavage of CD62L peptide by ADAM metalloproteinases, we included control cleavage of CD62L by recombinant ADAM10 in the presence and absence of the ADAM10 inhibitors, BB-94 and GI254023X (Supplemental Figure 1A). In addition, we included in the revised manuscript the detection of soluble CD62L in the supernatant on day 6 of post HIV infection in the presence and absence of BB-94 (Figure 1A). The concentration of soluble CD62L was significantly higher in day 6 infected than uninfected supernatants (both were stimulated with anti-CD3 for two days prior to infection). BB-94 partially reduced the soluble CD62L concentration. Interestingly, Vassena et al detected the loss of CD62L surface expression during HIV infection but concluded that the loss was due to Vpu and Nef sequestration of CD62L (Vassena et al. JVI 2015). We do not dispute the sequestration model but our previous publication also supports CD62L shedding (Kononchik et al. Nat Commu 2018). Both processes result in the loss of CD62L expression. It is likely that both sequestration and shedding of CD62L occur during HIV-1 infections. In the revised manuscript, we included a detection of soluble CD62L in day 6 infected supernatants in the presence of metalloproteinase inhibitor BB-94 (Figure 1). Higher CD62L concentrations were found in both infected samples than the uninfected sample in the day 6 supernatants. BB-94 partially reduced the soluble CD62L in the infected samples. The soluble CD62L detected by ELISA from Vassena et al’s publication showed less soluble CD62L presence in the HIV-1 infected than uninfected samples. We think the difference between Vassena’s and our ELISA results may be due to the experimental protocols. It is possible that SEB treatment resulting in more CD62L shedding than HIV-1 infection. We stimulated T cells prior to HIV infections and removed anti-CD3 before infections. As FACS data indicated that majority of CD62L expression loss occurred late in the viral infection, we limited the soluble CD62L measurement to only day 6 of post infection supernatants. To further reflect the reviewer’s comment, we revised the text to replace “CD62L shedding” with “loss of CD62L expression” throughout the manuscript for all data associated with FACS.

It is not clear to us how HIV-1 infection upregulates caspases expression. Others have published about the expression of procaspases under the control of inflammatory pathway, such as NF-kB etc. We measure the transcription of caspases by both RNAseq and RT-PCR as an indication of potential involvement of caspases in HIV-1 infection. Importantly, we measured activation of caspases using FLICA staining, and linked the activation of caspases in HIV infection to Nef. As to the mechanism of how caspases are activated by nef, it is an open question that requires further investigation. Our transcriptome analyses on nef+ or nef- HIV infected CD4 T cells suggest nef upregulate genes involved in multiple apoptotic pathways, including IFN-I, TNF-a, inflammasome and p53-mediated pathways (Figure 6A).

*Reviewer 1: Is the benefit of activating caspases(=enhanced release) larger than the drawbacks (=cell death)?*

Response*:* We think the benefit of activating caspases for HIV is larger than causing the death of infected cells. We would also reason that the way for infected cells to stay live is to become latent and not releasing viruses. The primary function of productively infected cells is to release viral progeny. Viral release alone may outpace membrane biosynthesis and leading to cell death—HIV is a lytic virus. Thus, the death of productively infected cells occurs after viral release and may not negatively impact viral transmission.

*Reviewer 1: Is shedding of CD62L just a side effect of cells undergoing apoptosis, similar to PARP cleavage?*

Response: It is likely that shedding of CD62L is one of many consequences of cells undergoing apoptosis, similar to PARP cleavage by activated caspases. The difference is that CD62L is not cleaved by caspases but rather cleaved by ADAM family of MMP.

*Reviewer 1: Please confirm results obtained with the pan-caspase inhibitor by performing caspase KO or KD (e.g. by CRISPR-Cas9 KO or siRNA transfections), or does pooling of the individual caspase inhibitors have the same impact as the pan-caspase inhibitor?*

Response: We did not perform caspase KO or KD on PBMC out of the concern that these experiments are not technically feasible. Primary PBMC are not susceptible for transfections in our hand. We had previously attempted to KD CD62L on PBMC with siRNA using lentiviral transductions to address the involvement of CD62L in HIV infection (Kononchik et al. Nat Commun 2018). The KD of CD62L in PBMC resulted in at best 20% reduction in cell surface CD62L expression FACS typing. To circumvent the technical difficult in genetic manipulation of primary T cells, we selected an HIV susceptible CD4 cell line, CEM and were able to manipulate its surface CD62L expression through transfection and KD by CRISPR (Kononchik et al. Nat Commun 2018). However, caspases appear to be only required in HIV infection of primary T cells but not cell lines like CEM as QVD-OPH had no effect in the viral infection of CEM. This was reported in Supplemental Figure 5G) of the original PLOS ONE submission. This was part of the evidence led us to probe the link between nef and caspase activation as nef is also dispensable in HIV infection of cell lines. Therefore, KO or KD caspases in CEM would not be informative. As to pooling of individual caspase inhibitors, we think, in principle, the combination should work providing all classes of caspases are suppressed in every cell. However, the cytotoxicity of the pooled inhibitors may be problematic to cells as the required aggregated inhibitor concentration to inhibit all caspases is likely higher than that of a single pan-caspase inhibitor. To address this, we performed additional experiments in which HIV infected PBMC were treated with 50uM belnacasan, 50uM DEVD, or their combination, and compared the results with 50uM QVD treated infections. The combined treatment inhibited HIV infection slightly more than their single treatments but significantly less than QVD-treated infections. It may require more than two specific caspase inhibitors to match the activity of a pan-caspase inhibitor. The new data is included as Supplemental Figure 4A-B.

*Reviewer 1: I do not see the paradigm shift yet the authors claim in their abstract. Would larger quantities of virus produced upon caspase-kick really outcompete a loss in replication/virus reservoir due to cell death? One major part of the kick and kill approach is to identify and activate dormant virus to selectively target it. How would that be impacted by caspase inhibitors and the proposed model and why should this study challenge that approach – i.e. change the current concept that released virus is easier to target than latent virus?*
Response: The paradigm shift refers to the opposite role of caspases in HIV infection and the treatment strategy. The current paradigm assumes HIV-1 infection-induced caspases activation is part of host anti-viral response to kill infected T cells, thus detrimental to the viral expansion. This belief in the role of caspases prompted a treatment strategy to stimulate procaspase8 expression and enhance apoptosis of latency reversal agents (LRA-kick) reactivated viral reservoir cells, as some of the stimulated viral reservoir cells resisted apoptotic cell death. However, our findings showed that viral release required caspase activation. Thus, caspases activation is not detrimental but potentially beneficial to the viral expansion. This shift in the role of caspases in HIV infection highlights a potential risk of stimulating caspases as a treatment strategy, which may cause more virus to release and potentially generating freshly infected reservoirs. Instead, because caspase inhibitors suppressed both productive and viral reservoir release (Figure 7A-B), we speculate the inhibition of caspases may reduce viral set points in infected individuals under ART regiment, as the residual viremia under ART is likely the result of an equilibrium between viral release from latent reservoirs and ART removal of released viruses. We think the amount of released virus may outcompete the benefit of causing reservoir cells to die based on observations that ex vivo stimulated viral reservoir cells resulted in more viral release and caspase inhibition in these experiments resulted in less viral release (Figure 7). If the death of stimulated viral reservoir cells preceded viral release (namely the benefit in viral release do not outcompete the death of reservoir cells), then inhibition of caspases should lead to more but not less released viruses. Our results suggest that viral release likely preceded cell death. We revised the discussion section to include this.

*Reviewer 1: Minor issues:
- Be more consistent with the cell type used in different assays and motivate the choice of the specific cell type. (Some results are shown for PBMCs only while others are specific for CD4 T cells or CD8, why?)*

Response: We always perform HIV infections using CD8-depleted PBMC. For the convenience of description, we simply use ‘PBMC’ in the results section whenever describing infections. But the CD8-depletions are clarified in the methods section. No CD8 T cells were specifically used in this manuscript. For RNAseq, additional steps were performed to enrich CD4 T cells for obvious reason to exclude none-relevant cells, such as B cells, monocytes etc in RNAseq analysis. We use ‘CD4+ T cells’ in describing FACS results where CD4 T cells were identified through gating. We also use ‘CD4+ T cells” when describing electron microscopy images as the images are obviously from infected CD4 T cells.

*Reviewer 1:- Please control for the infectivity of viral stocks (e.g. WT vs Nef- viruses), as Nef defective viruses often are -dependent on the setting- less infectious.*

Response: Nef is primarily required for HIV dissemination in primary CD4 T cells but dispensable in infection of T cell lines. We used a CD4 T cell line CEM to show the infection of WT(Nef+) and Nef- viruses to CEM cell line are near identical. This data was in Supplemental Figure 5G.

*Reviewer 1:- Fig. 2D-E: perform the FACS stainings in the same cells!*

Response: The bar diagrams of Figure 2D-E are derived from multiple experiments of FACS staining of the same cells shown in 2A-B. The FACS plots in 2A-B are one representative of the independent experiments.

*Reviewer 1:- Replica of experiments to have n=3 donors or independent experiments: e.g. 1F/4E/ (was only one donor in duplicates or triplicates), 6A (two donors)*

Response: Most of the experiments presented were performed from at least two independent donors.

*Reviewer 1:- Include Viability assay to exclude cytotoxic effect of the different compounds on the primary cells. E.g. for using DMSO and the pan-caspase inhibitor QVD-OPH.*

Response: Viability assay of cells treated with titration amount of QVD-OPH vs DMSO is presented in Supplemental Figure 4C of the original submission.

*Reviewer 1:- Please optimize the figure legends: Including steps of the method which are important to understand the result.*

Response: Similar comments were raised from reviewer 2 and we have extensively revised all figure legends according to the comment.

*Reviewer 1: - Please improve the labeling of the figures; e.g. for the Western Blot in Fig. 4F the sizes of the bands are missing and for Fig. 5 the scale bar is missing as well as labelling above about the origin of the picture (TEM/EM)*

Response: The molecular ladder is added the revised western blot, now named as Figure 5I. The scale bars are included in Figure 5 and TEM and SEM are stated in Figure legends.

*Reviewer 1:- Fig 5D: Can the authors identify the structures labeled with yellow arrows beyond claiming they could be virus-like particles? How would HIV stuck to the surface by shedding resistant CD62L look like?*

Response: Further identifying and isolating the particles pointed by yellow arrows is a subject of our on-going research. It is not trivial to identify them as there is no prior reports to our knowledge documenting the technical approach. As to using shedding resistant CD62L to exam the viral budding by EM, the predicted phenotype would be similar to cells treated with BB-94 or caspase inhibitors in that budding virions may be tethered by CD62L similar to that observed in Figure 5F. While mouse shedding resistant CD62L was reported, it is not clear if similar mutations renders human CD62L resistant to shedding as no human CD62L shedding resistant mutations are reported. In addition, primary PBMC are less amendable for transfection. We thank for the reviewer for the comment. The work, however, is quite substantial and beyond the scope of this manuscript.

*Reviewer 1:-* *Overview figure needs to be improved (!): labelling should be Casp and not Cas, the mitochondria is not identifiable. Why does it show HIV-1 entry by endocytosis in a double-layered vesicles?*

Response: the figure has been revised according to the comments. As some caspases are associated with mitochondria and some are cytosolic, we removed the drawing of mitochondria designation. The current overview figure simply indicates caspases leading to ADAM activation and shedding of CD62L.

*Reviewer 1:-* *Please show for the FACS data (Fig. 2A) single stainings*

Response: We assume the reviewer meant one dimensional histogram display instead of 2-dimensional contour plots, we included the corresponding histograms in Supplemental Figure 3E.

*Reviewer #2: The manuscript proposes a novel and interesting role of caspases in the life cycle of HIV-1, especially important in primary CD4+ cells. I do not have major objections about the experiments and I find the results intriguing. Having read the manuscript, the authors convinced me that the requirement of caspases activity for the release and infectivity of HIV is certainly a concept that deserves more attention and further experiments that this manuscript will certainly stimulate.
However, this is a very dense and massive manuscript which requires an effort to follow. My only request is that authors would make it easier for the readers to follow and understand the experiments, especially by paying attention to the way they formulate the figure legends. Most figure legends are written as if they were a summary of the results, rather than taking care of providing the technical information necessary to understand what kind of data the various graphs represent. Here are some suggestions, but I encourage the authors to revise all legends accordingly.*

Response: We thank for the reviewer for supportive comments and have extensively revised all figure legends both in the manuscript and in the supplemental information according to the comments.

*Reviewer 2: Figure 1A. Please state the meaning of UI in the legend. Also it is not clear what “Results are pooled from duplicates of conditions from two independent experiments” means in terms of how statistic was assessed. How was t-test calculated from technical or biological repeats? Please explain.*

Response: UI stands for uninfected. Figure 1A legend is revised accordingly. The t-test was performs on technical repeats for each experiment. In general, we do not combine data from different experiments in statistical analyses as significant variations are found between donors and between different infection levels.

*Reviewer 2: Figure 3. The legend is a succinct result section rather than an explanation of what the data shown in the figure are and how they were acquired. I need to be able to understand what experimental technique was used. I assume that these are data mostly from flow cytometry. It is important to say it, to briefly explain what the data are, which technique was used to acquire them.*

Response: the figure legend is revised accordingly.

*Reviewer 2: Figure 4:
For fig 4A see same comment as figure 3
Fig 4C: can you explain the normalization? Are these copies of viral RNA normalized per ug of gDNA or what else?*
*Fig 4E. Relative gene expression: relative to what? The reader needs to know.*

Response: Figure legend is revised. The viral RNA are normalized to 10^6^ cells. The relative gene expression level in Figure 4E is calculated relative to that of beta actin. The figure legend is revised to clarify this.

*Reviewer 2: Figure 5H: “Data taken from triplicate conditions per dose for each treatment and is representative of two independent experiments”. This is really difficult to understand. So, this is a representative experiment and the statistics are on technical replicates?*

Response: The data were presented as triplicates from each treatment and the statistical analysis was done on the technical triplicates using 2-way ANOVA test *p < 0.05, **p < 0.01, ****p < 0.0001. The results are representative of two independent experiments. The figure legend is revised accordingly.

*Reviewer 2: Figure 6A: please state if this is from RNAseq or RT-PCR*
Response: Figure legend is revised to provide description on RNAseq analysis.

*Reviewer 2: Figure 7. X axis: "days" from what?
Y axis:. Virion associated HIV RNA. How have the authors ensured that this RNA is virion-associated? Were virions pelleted on sucrose to enrich for viral particles? Would it be more appropriate to label the axis “released HIV RNA”?*

Response: X-axis is days of anti-CD3 stimulation. Y axis label has been revised to HIV RNA (copies/ml).

In an effort to improve clarity of the presentation, we removed some partially redundant supplemental figures. They include the previous Supplemental Figure 5C, a representative set of FACS contour plots from which the bar-diagrams in the revised Supplemental Figure 5C,5D are derived. We also removed Supplemental Figure 6 and 7, that were included as an appendix to address a previous reviewer’s comment.
